# Supplementary figures and images for: Agility Testing in Youth Football (Soccer)Players; Evaluating Reliability, Validity, and Correlates of Newly Developed Testing Protocols
Source: Int J Environ Res Public Health. 2020 Jan 1;17(1):294. doi: 10.3390/ijerph17010294 (PMC6981745; doi:10.3390/ijerph17010294)

## Supplementary figure S1. Distributions of the fitness variables

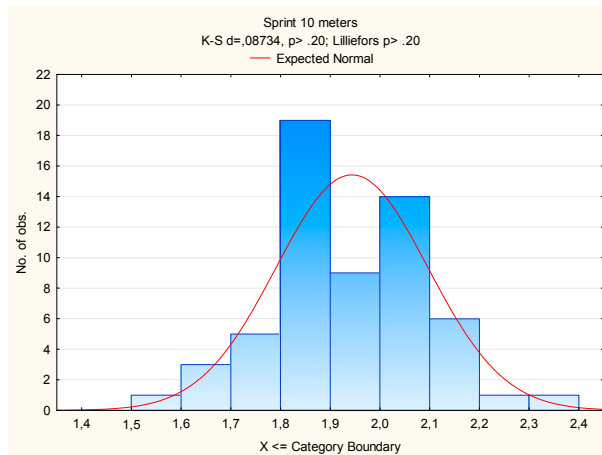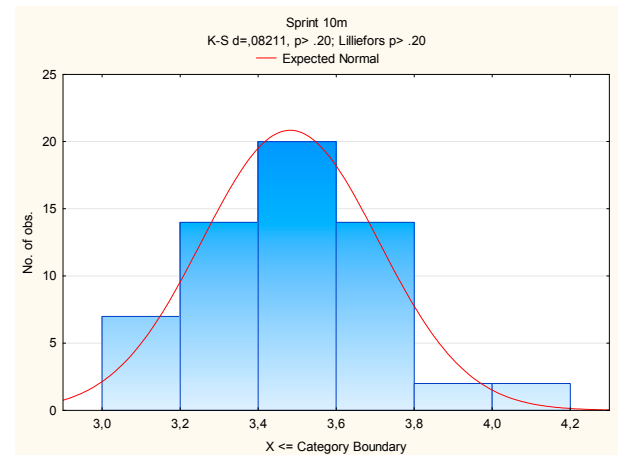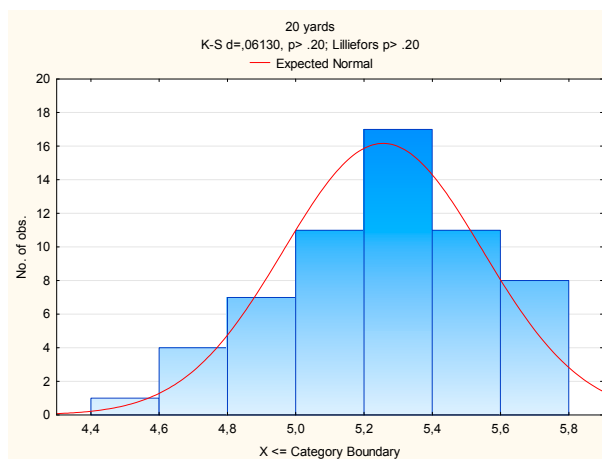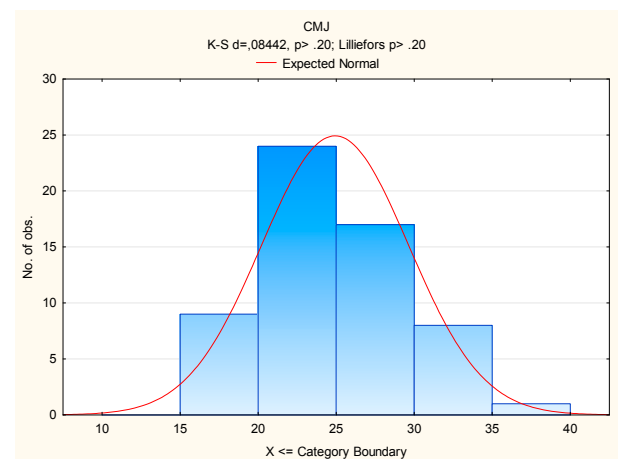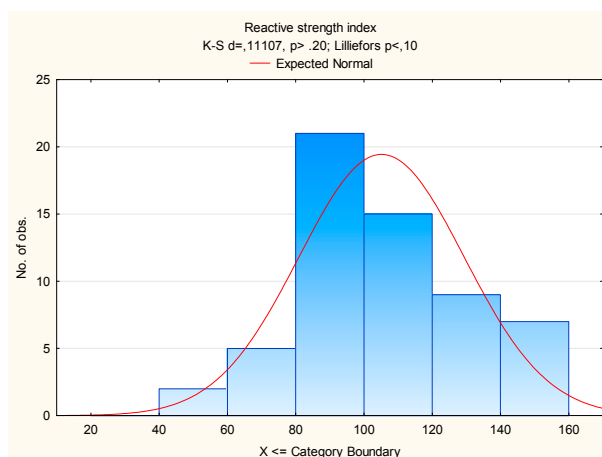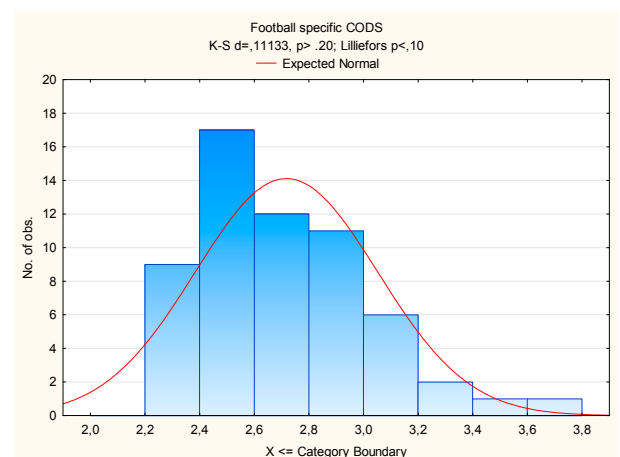

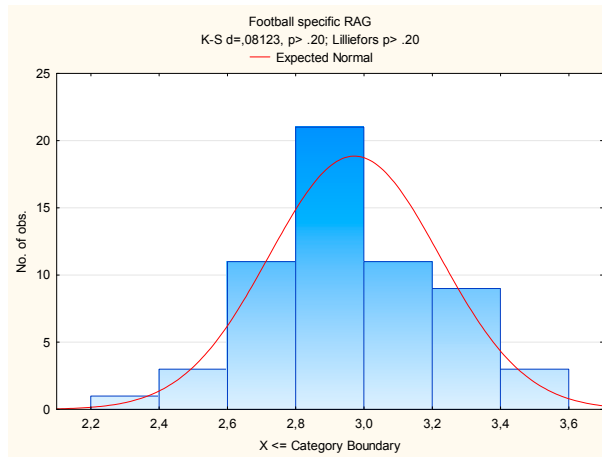

Supplement: Supplementary file 1 [file ijerph-17-00294-s001.pdf]
